# Evidenzbasierung in Primärprävention und Gesundheitsförderung: Methoden und Vorgehensweisen in 5 Forschungsverbünden

**DOI:** 10.1007/s00103-021-03322-z

**Published:** 2021-04-09

**Authors:** Mirko Brandes, Saskia Muellmann, Theresa Allweiss, Ulrich Bauer, Andreas Bethmann, Sarah Forberger, Jennifer Frense, Peter Gelius, Klaus Pfeifer, Orkan Okan, Britta Renner, Harald Schupp, Michael Wright, Hajo Zeeb

**Affiliations:** 1 Abteilung Prävention und Evaluation, Leibniz-Institut für Präventionsforschung und Epidemiologie – BIPS, Achterstr. 30, 28359 Bremen, Deutschland; 2 Katholische Hochschule für Sozialwesen Berlin, Berlin, Deutschland; 3 Fakultät für Erziehungswissenschaft, Zentrum für Prävention und Intervention im Kindes- und Jugendalter (ZPI), Interdisziplinäres Zentrum für Gesundheitskompetenzforschung (IZGK), Universität Bielefeld, Bielefeld, Deutschland; 4 Department für Sportwissenschaft und Sport, Friedrich-Alexander-Universität Erlangen-Nürnberg, Erlangen, Deutschland; 5 Fachbereich Psychologie, Universität Konstanz, Konstanz, Deutschland; 6 Wissenschaftsschwerpunkt Gesundheitswissenschaften, Universität Bremen, Bremen, Deutschland

**Keywords:** Primärprävention, Gesundheitsförderung, Evidenzbasierung, Studiendesign, Implementierung, Primary prevention, Health promotion, Evidence-based public health, Study design, Implementation

## Abstract

Von 2014 bis 2022 erforschen die 5 deutschen Forschungsverbünde AEQUIPA, CAPITAL4HEALTH, HLCA, PartKommPlus und SMARTACT Themen der Primärprävention und Gesundheitsförderung mit dem Ziel, die Evidenzgrundlagen in diesen Bereichen weiterzuentwickeln. In diesem Beitrag wird die Arbeit der 5 Forschungsverbünde für Primärprävention und Gesundheitsförderung unter dem Aspekt der Evidenzbasierung aus der internen Perspektive vorgestellt, analysiert und diskutiert. Als orientierender Rahmen dient ein Modell der evidenzbasierten Public Health.

Die 5 Forschungsverbünde nutzen für die Evidenzgenerierung vielfältige Zugangswege bzgl. der Beteiligung nichtakademischer, zivilgesellschaftlicher Akteur*innen und Nutzer*innen. Es finden sich vielfältige Studiendesigns, die von randomisiert kontrollierten Studien und systematischen Reviews zu diversen qualitativen Designs reichen. Die Nutzung von Modellen und Theorien unterstützt die Evidenzbasierung. Über die Evidenzentwicklung hinaus legen alle Verbünde einen Schwerpunkt auf die zumindest exemplarische Implementierung des neuen Wissens.

Durch die Methodenvielfalt kann eine breit gefächerte Evidenzbasierung unter Berücksichtigung verbundspezifischer Aspekte realisiert werden. Grenzen für eine weitere systematische Stärkung der Evidenzbasierung liegen in strukturellen Rahmenbedingungen. Insbesondere die Einbindung von nichtakademischen, zivilgesellschaftlichen Akteur*innen und Nutzer*innen für die Arbeit mit schwer erreichbaren Zielgruppen kann oft nicht ausfinanziert bzw. zeitlich berücksichtigt werden. Die COVID-19-Pandemie verdeutlicht die Wichtigkeit eines flexiblen Methodenspektrums, in dem ein sinnvolles Zusammenspiel von digitalen und analogen Methoden anzustreben ist.

## Abstract (en)

Between 2014 and 2022, the 5 German research networks AEQUIPA, CAPITAL4HEALTH, HLCA, PartKommPlus, and SMARTACT are investigating topics of primary prevention and health promotion with the aim of further deepening the evidence base in these areas. The work of the 5 research networks for primary prevention and health promotion is presented, analysed, and discussed from an internal perspective. A model of evidence-based public health serves as a structuring framework.

The 5 research networks use a variety of access routes for the generation of evidence with regard to the participation of nonacademic, civil society actors and users. There is a wide range of study designs – from randomised controlled trials and systematic reviews to diverse qualitative designs. The use of models and theories supports the evidence base. Beyond evidence generation, all research networks focus on at least exemplary implementation of new evidence.

Due to the diversity of methods, a diversified evidence-based approach can be realised, taking into account network-specific aspects. Structural circumstances limit the further systematic strengthening of the evidence base. In particular, the involvement of nonacademic, civil society actors for the work with hard-to-reach target groups often cannot be financed or is considered too time consuming under the given circumstances. The COVID-19 pandemic highlights the importance of a flexible spectrum of methods, employing both digital and analogue methods in a meaningful way.

## Einleitung

Das Bundesministerium für Bildung und Forschung fördert von 2014 bis 2022 durchgängig 5 Forschungsverbünde, die prioritäre Themen der Primärprävention und Gesundheitsförderung erforschen. Zu den Zielen der Verbünde gehört, die Evidenzgrundlagen für Präventions- und Gesundheitsförderungsmaßnahmen in den jeweiligen Themenschwerpunkten zu verbessern sowie die Umsetzung von Maßnahmen systematisch zu unterstützen und zu evaluieren (Tab. 1). Eine kompakte Darstellung der einzelnen Verbünde sowie Verweise auf detailliertere Informationen sind auf der Webseite des Forschungsnetzwerkes Primärprävention und Gesundheitsförderung www.fp2g.net zu finden.

**Table 1:** 

Kürzel	Offizieller Verbundtitel	Ort und Verbundleitung	Themen
AEQUIPA	Körperliche Aktivität, Gerechtigkeit und Gesundheit: Primärprävention für gesundes Altern	Bremen (Prof. Dr. H. Zeeb)	Bewegung als Schlüsselfaktor für gesundes Altern
Capital4Health	Handlungsmöglichkeiten für aktive Lebensstile: Ein Forschungsnetzwerk für interaktiven Wissensaustausch in der Gesundheitsförderung	Erlangen (Prof. Dr. K. Pfeifer)	Nachhaltige Förderung aktiver Lebensstile in realweltlichen Settings
HLCA	Health Literacy in Childhood and Adolescence	Bielefeld (Prof. Dr. U. Bauer)	Gesundheitliche Grundbildung bei Kindern und Jugendlichen
PartKommPlus	Forschungsverbund für gesunde Kommunen	Berlin (Prof. Dr. M. Wright)	Gesunde Kommunen durch integrierte, partizipative Strategien der Gesundheitsförderung
SMARTACT	Individuelle und kontextbasierte Interventionen in Echtzeit zur Förderung des normalen Essverhaltens und der körperlichen Aktivität unter Einsatz mobiler Technologie	Konstanz (Prof. Dr. B. Renner)	Verbesserung des Gesundheitsverhaltens mithilfe des Einsatzes mobiler Technologien

Mit den genannten – und weiteren verbundspezifischen – Zielen lässt sich die wissenschaftliche Arbeit der Präventionsforschungsverbünde einem Verständnis von evidenzbasierter Public Health (EBPH) zuordnen, das EBPH als Prozess der Integration wissenschaftsbasierter Interventionen und Präferenzen der jeweiligen Community mit dem Ziel der Verbesserung der Gesundheit der Bevölkerung [1] definiert. Offensichtlich ist, dass anders als in der evidenzbasierten Medizin nicht die individuellen Patient*innen im Mittelpunkt stehen, sondern die Gesundheit auf Bevölkerungsebene. Die Berücksichtigung interdisziplinärer Erkenntnisse bei Begründung, Auswahl und Entwicklung von Maßnahmen ist ein weiteres Charakteristikum von EBPH. Dies ist verbunden mit einem systematischen und transparenten Vorgehen im Prozess der Entscheidungsfindung und -umsetzung [2], das sich in seinen Grundprinzipien an die evidenzbasierte Medizin anlehnt [3].

**Figure 1:**
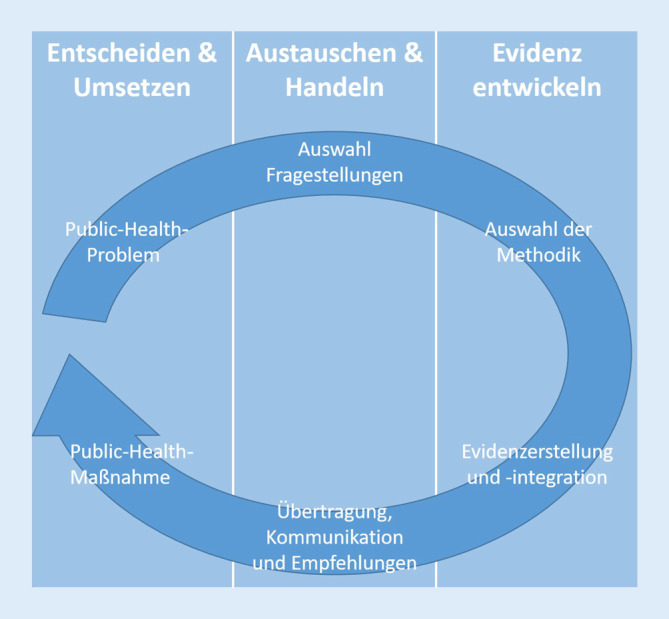

Evidenzbasiertes Vorgehen umfasst nach dem Modell von Gerhardus (2010) das Durchlaufen der Bereiche „Entscheiden und Umsetzen“, „Austauschen und Handeln“, und „Evidenz entwickeln“ (Abb. 1; [2]). Der Bereich *Entscheiden und Umsetzen* steht dabei am Anfang und am Ende des Prozesses und umfasst sowohl die Entscheidung, welches Public-Health-Problem untersucht werden soll, als auch die Entscheidung, ob auf Basis des Forschungsprozesses z. B. die zuvor entwickelte und evaluierte Public-Health-Intervention durchgeführt wird. Im mittleren Bereich, *Austauschen und Handeln*, wird das Public-Health-Problem in wissenschaftlich zu untersuchende Fragestellungen überführt bzw. es werden anhand der im Forschungsprozess gewonnenen Erkenntnisse konkrete Handlungsempfehlungen abgeleitet. Im Bereich *Evidenz entwickeln *erfolgen die Auswahl der am besten geeigneten Methoden zur Beantwortung der Fragestellungen sowie die Durchführung des Forschungsprozesses mit den ausgewählten Methoden.

Beim Durchlaufen der genannten Bereiche ist zu betonen, dass evidenzbasiertes Vorgehen in Prävention und Gesundheitsförderung nicht nur im Verantwortungsbereich von Wissenschaftler*innen liegt, sondern die intensive Beteiligung von Stakeholder*innen einschließt. Die Einbindung von Stakeholder*innen ist in allen 3 Bereichen in unterschiedlicher Form umsetzbar. Im Bereich „Entscheiden und Umsetzen“ bietet sich partizipatives Vorgehen besonders an, um relevante Public-Health-Probleme zu identifizieren bzw. Public-Health-Maßnahmen angemessen und adressatenspezifisch umzusetzen.

Bei der Auswahl der Methoden zur Evidenzentwicklung ist zu beachten, dass sowohl die Adressatengruppen und Settings als auch die Maßnahmen in der Primärprävention und Gesundheitsförderung sehr vielfältig sind. Das Herzstück der evidenzbasierten Medizin, randomisierte kontrollierte Studien (RCT), bildet daher nur einen kleineren Teil des verfügbaren und benötigten Methodenspektrums der EBPH [4, 5]. Die 5 Forschungsverbünde spiegeln die Diversität und Breite sowohl in Hinsicht auf den Zugang als auch die Methoden und die Umsetzung eines evidenzbasierten Vorgehens in der Forschung zu Prävention und Gesundheitsförderung wider.

Wir beschreiben und diskutieren im Folgenden die Methodenvielfalt und die Vorgehensweisen der Forschungsverbünde orientiert am Prozess des evidenzbasierten Vorgehens nach dem EBPH-Modell von Gerhardus (Abb. 1; [2]). Dabei erläutern wir jeweils kurz die inhaltlichen Kernthematiken der von den Autor*innen koordinierten Verbünde und gehen dann – aufgrund der Diversität der Verbünde unterschiedlich akzentuiert – auf Aspekte der Evidenzbasierung ein.

## Beispiele und Vorgehensweisen der Evidenzbasierung in den Forschungsverbünden

### AEQUIPA

Der Forschungsverbund *AEQUIPA* (Körperliche Aktivität, Gerechtigkeit und Gesundheit: Primärprävention für gesundes Altern) untersucht in 5 Teilprojekten Maßnahmen und Strategien zur primärpräventiven Bewegungsförderung bei älteren Menschen. Gesundheitliche Chancengleichheit ist als Querschnittsthema angelegt. Dabei nutzt *AEQUIPA* das sozialökologische Modell von Sallis [6], um sowohl auf die individuellen (z. B. gesundheitsbezogenes Verhalten) als auch kontextuellen Faktoren (z. B. Umwelt- und Sozialbedingungen) einzugehen, die für Bewegung bei Älteren förderlich oder hinderlich sind [7].

Die Forschungsarbeiten in AEQUIPA sind in der Systematik des EBPH-Modells allen 3 Bereichen in unterschiedlichem Maße zuzuordnen. Das Public-Health- bzw. Gesundheitsförderungsproblem „Bewegung und gesundes Altern“ wurde aus wissenschaftlicher Literatur sowie gesundheitspolitischen Situationsbeschreibungen abgeleitet, die konkreten Fragestellungen durch eine systematische Aufbereitung der Informationen über körperliche Aktivität als wesentlicher Baustein für gesundes Altern entwickelt. Insbesondere für die zweite Förderphase des Verbundes konnten die bereits im Netzwerk vorhandenen Stakeholder*innen (z. B. Vertreter*innen der Metropolregion Nordwest, Krankenkassen) in die Entwicklung der weiteren Fragestellungen einbezogen werden, zugleich wurden Präferenzen der Studienteilnehmenden verstärkt berücksichtigt.

Die Methoden der Evidenzgenerierung umfassten sowohl die Entwicklung und Evaluation individueller Bewegungsinterventionen für ältere Erwachsene als auch strukturelle und systemische Ansätze mit dem Fokus auf Ressourcen oder auf der Entwicklung und Analyse förderlicher Strukturen auf Gemeindeebene [8–10]. So wurden kooperative Ansätze zur Indikatorenentwicklung mit Akteur*innen aus den Bereichen Stadt- und Verkehrsplanung, (öffentliche) Gesundheit, Umwelt unter Einbeziehung von Senior*innen als direkte Adressaten durchgeführt. Der Folgeschritt von der Evidenzgenerierung zur Übertragung und Empfehlungsentwicklung, konkret die Integration der gefundenen Indikatoren in den Planungsprozess, wurde ebenfalls unter Nutzung von Expert*inneninterviews wissenschaftlich analysiert [9]. Dies ist ein Hinweis darauf, dass in der konkreten Forschungspraxis Erkenntnisentwicklung und Evidenzgenerierung auf unterschiedliche Weise in allen Bereichen des EBPH-Modells stattfinden und sich so u. a. Schnittstellen zur Implementationsforschung [11] ergeben.

Für die Evidenzgenerierung in AEQUIPA kamen randomisierte kontrollierte Studien zur Effektermittlung bei Bewegungsinterventionen [12] ebenso zum Einsatz wie Verfahren der interdisziplinären Technologietestung. Hier wurden qualitative und quantitative Interviews hinsichtlich Nutzer- und Technologieakzeptanz durchgeführt und die Heterogenität möglicher Nutzer*innen konkret als Forschungsgegenstand thematisiert [13]. Evidenzsynthese als typischer Ansatz der EBPH erfolgte in systematischen Übersichtsarbeiten (z. B. Czwikla et al. [14]) und einer Folgenabschätzung zur gesundheitlichen Chancengleichheit [15], die eine Modellierung und Quantifizierung erwarteter Gesundheitseffekte durch bewegungsförderliche Interventionen bei Älteren beinhaltete. Gesundheitliche Chancengleichheit wurde ebenfalls mittels Modellierung von Szenarien zu sozialen Unterschieden bei der Untersuchung der Interventionswirksamkeit aufgegriffen, wodurch konkrete Hinweise für eine angemessene Umsetzung der Erkenntnisse in Public-Health-Maßnahmen erarbeitet wurden [15]. Insgesamt konnte durch die intensive Abstimmung im Verbund ein Vorgehen umgesetzt werden, das die Komplexität der Förderung körperlicher Aktivität bei Älteren berücksichtigt und ein gemeinsames Lernen von Forschenden und Akteur*innen ermöglicht. Im Sinne des EBPH-Modells lagen die Arbeitsschwerpunkte vornehmlich in Bereich der Evidenzentwicklung und im Austauschen und Handeln.

### Capital4Health

Der Verbund *Capital4Health* (Handlungsmöglichkeiten für aktive Lebensstile: Ein Forschungsnetzwerk für interaktiven Wissensaustausch in der Gesundheitsförderung) nutzt partizipative Ansätze, um in verschiedenen Settings (Kitas, Schulen, berufliche Bildung, Gemeindesetting) unter Einbeziehung relevanter Gruppen und Organisationen Maßnahmen zur Bewegungsförderung zu entwickeln, zu implementieren und zu evaluieren. Ähnlich wie *AEQUIPA* widmet sich *Capital4Health* besonders den EBPH-Bereichen der Evidenzentwicklung und des Handelns/Austausches, wobei die Kombination von verschiedenen Arten von Evidenz charakteristisch für die Arbeit im Verbund ist. Das Konsortium ist sich der Grenzen einer rein wissenschaftlichen Evidenzbasierung bewusst: Rein akademisch bzw. unter Laborbedingungen durchgeführte Interventionen bleiben oft in der Demonstrationsphase stecken [16]; trotz guter Evidenzen für die Wirksamkeit gelingt die praktische Umsetzung in der Fläche nicht, z. B. aufgrund geringer Akzeptanz durch Zielgruppen, geringer Passfähigkeit auf die Rahmenbedingungen des Settings oder mangelnder politischer Unterstützung.

Zentral für die Konzeption des Gesamtverbundes sowie der einzelnen Projekte war die wissenschaftliche Evidenz zu Bewegung und Gesundheit [17, 18], der Handlungsmöglichkeiten [19, 20] und der transdisziplinären Forschung [21]. Der Verbund arbeitet fortlaufend an der Erweiterung dieser wissenschaftlichen Evidenzgrundlagen, u. a. durch systematische Übersichtsarbeiten [22–24], empirische Erhebungen zur Wirksamkeit der durchgeführten Interventionen [25–28] sowie durch Beiträge zur Weiterentwicklung der theoretischen Grundkonzepte [29, 30]. Die Beiträge zur Theorieentwicklung verweisen dabei auf eine nicht immer beachtete Akzentuierung von EBPH: Nicht nur die geprüfte Anwendung von Konzepten, sondern auch die Erweiterung des theoretischen Rahmengerüstes von Public Health samt der empirischen Überprüfung neuer Theorien machen EBPH aus.

*Capital4Health* betreibt transdisziplinäre Forschung, bei der relevante Gruppen und Organisationen gemeinsam arbeiten, um relevantes, auf das Setting angepasstes Wissen zu produzieren [21, 29]. Dazu zählen Vertreter*innen der Zielgruppen, Multiplikator*innen (z. B. Expert*innen, Lehrkräfte und Vertreter*innen zentraler Organisationen der Gesundheitsförderung) und politische Entscheidungsträger*innen. Der Verbund nutzt den Ansatz der kooperativen Planung [31], der einen strukturierten, zielgerichteten Planungsprozess durch eine ausgeglichen besetzte Planungsgruppe vorsieht, um Maßnahmen zur Bewegungsförderung zu entwickeln. Insgesamt wurden vom Verbund 22 Planungsprozesse mit insgesamt 144 Sitzungen durchgeführt, an denen im Schnitt je 9 Beteiligte teilnahmen [32]. Das so koproduzierte Wissen mündete in ein breites Spektrum an Maßnahmen, das von einzelnen Unterrichtseinheiten zu Bewegung und Gesundheit bis hin zu Infrastrukturmaßnahmen und bayernweiten Lehrplanänderungen reichte.

Für die Schritte der Evidenzerstellung und -integration in Bezug auf die ausgewählten Interventionen nutzt *Capital4Health* ein breites Methodenspektrum der qualitativen und quantitativen Forschung. Die bisherigen Evaluationsergebnisse legen nahe, dass durch den transdisziplinären Ansatz Wirkungen auf verschiedenen Ebenen erzielt werden konnten, z. B. die Schaffung individueller Kompetenzen, der Aufbau organisationaler Kapazitäten und die Steigerung der Schrittzahl bei Kindern und Kitabetreuerinnen [25–28]. Insgesamt zeigen die Erfahrungen und Ergebnisse aus *Capital4Health*, dass die primär wissenschaftliche Evidenz eine wichtige Basis und Zielgröße für die Forschung bleibt, dass aber die zusätzliche Nutzung des Wissens relevanter Gruppen und Organisationen für die Erzielung nachhaltiger Wirkungen unter realweltlichen Bedingungen zentral ist. Transdisziplinäre Ansätze und Methoden wie die kooperative Planung können hierzu einen wichtigen Beitrag leisten. Kritisch anzumerken ist, dass aufgrund der aktuellen Projektförderungslogik kaum Mittel zur Verfügung stehen, um nichtakademische Akteure bereits in die Entwicklung von Forschungsanträgen einzubinden, etwa bei der gemeinschaftlichen Definition des Public-Health-Problems und der spezifischen Fragestellungen. Dementsprechend folgen viele Projekte konzeptionell einer vorwiegend wissenschaftlichen Logik, was nur bedingt durch Partizipation in späteren Phasen ausgeglichen werden kann.

### HLCA

Der *HLCA*-Verbund (Health Literacy in Childhood and Adolescence) bearbeitet in den 20 Teilprojekten eine erhebliche Bandbreite von Fragestellungen zu Health Literacy bei Kindern und Jugendlichen [33]. Die verschiedenen Formen der Evidenzbasierung wurden jeweils von den vorgegeben Projektzielen und damit assoziierten methodischen Vorgehensweisen determiniert.

Die Evidenzentwicklung basierte analog zu den übrigen Verbünden auf qualitativen, quantitativen und zudem konzeptentwickelnden, vergleichenden Methoden, die stark interdisziplinär fundiert sind und Bereiche wie Theorie‑, Fragebogen‑, Interventions- und Politikforschung umfassten. Zudem wurde die Partizipation der Zielgruppen und relevanter Stakeholder*innen über verschiedene Analyse- und Befragungsmethoden sowie im Rahmen von Validierungsprozessen sichergestellt (z. B. Workshops, Interviews, Delphi-Befragungen). In den Projekten wurden narrative, systematische und metanarrative Literaturrecherchen und Synthesen durchgeführt, um theoretische Konzepte abzubilden [34], Messmethoden und Instrumente zu erfassen [35], die Evidenz und den Stand der Forschung abzubilden [36, 37], Vergleiche zwischen Ansätzen und Disziplinen herzustellen [38] und gesundheitspolitische Erkenntnisse des eigenen Forschungsbereichs zusammenzufassen [39]. Im Kontext des EBPH-Modells kann dieser Teil der Forschungsarbeiten als eigenständige Konzeptionierung des Themenfeldes Gesundheitskompetenz verbunden mit stark theoriegeleiteter Evidenzentwicklung eingeordnet werden. Die Erkenntnisse wurden verwendet, um eigene Modelle [40] und theoretisch-konzeptionelle Forschungsansätze zu begründen [41], Fragebögen zu entwickeln [42, 43] und empirische Studien durchzuführen [44–46], die als Evidenzbasis für eigene Interventionen dienten [47]. Untersuchungen der Wirkungsweisen und der Effektivität sowie gesundheitsökonomischer Aspekte von Förderprogrammen generierten neue Erkenntnisse zu Health-Literacy-Interventionen [48, 49].

Die Evidenzentwicklung ist eng verknüpft mit Schritten des Austauschens und Handelns, etwa in der Unterstützung der Replikation internationaler Programme an Schulen in Deutschland [50]. Neben den analytischen und empirischen Forschungsprozessen in den *HLCA*-Teilprojekten bilden der Forschungstransfer und die Ergebnissynthese die Strategie zur Validierung projektübergreifender Ergebnisse und für die Formulierung von Empfehlungen für Wissenschaft, Praxis und Politik. Die Synthese hat dabei die Aufgabe, die im Verbund erzeugten, wissenschaftlichen Erkenntnisse in Empfehlungen für die weitere Forschungs‑, Anwendungs- und Praxisorientierung sowie das Politikfeld zu übertragen. Der Verbund hat zunächst interne Empfehlungen für neue Forschungsschwerpunkte im Bereich der Gesundheitskompetenzforschung im Kindes- und Jugendalter hervorgebracht, die systematische Berücksichtigung in der Schule betont, Erkenntnisse für Professions- und Organisationsentwicklung erzeugt sowie über bildungs- und gesundheitsbezogene Politikstrategien informiert. *HLCA* trägt durch diese Schritte zur Umsetzung evidenzbasierter Public-Health-Maßnahmen zur Gesundheitskompetenz bei jungen Menschen bei.

### PartKommPlus

*PartKommPlus – Forschungsverbund für gesunde Kommunen *besteht aus 7 Teilprojekten, die im Rahmen von 11 Fallstudien in 8 Bundesländern Fragen der Partizipation in der kommunalen Gesundheitsförderung nachgehen. Jedem Teilprojekt liegt ein eigenständiger partizipativer Prozess der Entscheidung und Umsetzung zugrunde, sodass die konkreten Fragestellungen stark variieren. Einige Fragen beziehen sich beispielsweise auf die Entwicklung von Interventionen für bestimmte Bevölkerungsgruppen, während andere Fragen sich mit Methoden der Partizipation in der Forschung und Praxis der Gesundheitsförderung befassen.

Alle Teilprojekte haben den Ansatz der partizipativen Gesundheitsforschung (PGF) gemeinsam. In der PGF werden Personen, die von der Thematik der Forschung betroffen sind (z. B. Fachkräfte, Patient*innen, Adressat*innen von gesundheitsfördernden Maßnahmen), in den Forschungsprozess eingebunden. Sie gestalten das Forschungsvorhaben zusammen mit Wissenschaftler*innen aktiv mit, indem sie sich an den Entscheidungen über Forschungsfragen, die Art der Datenerhebung, die Interpretation der Ergebnisse und der Verbreitung der Ergebnisse beteiligen. Die PGF möchte nicht nur Gesundheitsprobleme und ihre Ursachen beschreiben und erklären, sondern auch den notwendigen sozialen Wandel zur Verbesserung der Situation herbeiführen [51]. Die Ziele der PGF sind es, einerseits neue Erkenntnisse zu generieren und andererseits Veränderungen zur Förderung von Gesundheit und Wohlbefinden anzustoßen sowie gesundheitliche Chancengleichheit zu stärken [52]. Daher sind die zentralen Fragen der Evidenz in der PGF, ob und wie der partnerschaftliche Forschungsprozess und die daraus resultierenden Erkenntnisse und Handlungen zu positiven Veränderungen im Sinne der Forschungsziele beigetragen haben. International werden diese Fragen unter dem Begriff Impact diskutiert, der nach Greenhalgh und Kollegen [53] als Nutzen, der über die Erzeugung wissenschaftlicher Erkenntnisse und Theoriebildung hinausgeht, definiert ist. Gemeint sind in diesem Zusammenhang die ökonomischen, umweltbezogenen, kulturellen, sozialen und gesundheitlichen Effekte von Forschung auf gesamtgesellschaftlicher Ebene, wozu in den Gesundheitswissenschaften ebenfalls die Verringerung gesundheitlicher Ungleichheit gehört. Innerhalb der PGF werden unter dem Begriff Forschungsimpact nicht nur beabsichtigte und unbeabsichtigte Veränderungen auf Makroebene (z. B. Gesellschaft, nationale Politik), sondern auch auf Meso- und Mikroebene (z. B. Kommunen, Institutionen und Individuen) subsumiert. *PartKommPlus* hat sich als Ziel seiner Arbeit gesetzt, den eigenen Forschungsimpact samt seiner Entstehungswege zu erfassen, zu beschreiben und online (siehe: www.partkommplus.de) zu veröffentlichen [54].

Eine Wirkungsbeschreibung („impact narrative“) wird für jedes Teilprojekt und für den gesamten Verbund erstellt. Je nach Teilprojekt dienen unterschiedliche qualitative und quantitative Datenquellen als Basis für die Wirkungsbeschreibung. Die Erhebung und Auswertung von Daten mit dem Schwerpunkt Wirkung erfolgen als Teil des partizipativen Forschungsprozesses vor Ort. Die Erstellung jeder Wirkungsbeschreibung wird von einer externen internationalen Expertin im Bereich Wirkung in der PGF begleitet und durch einen regelmäßigen Austausch unter den Teilprojekten und Beratungsangebote der Verbundkoordinierungsstelle unterstützt. Durch die Veröffentlichung dieser Beschreibungen und auch anderer Publikationen zum Thema Impact leistet *PartKommPlus* einen Beitrag zu Fragen der Erfassung, Darstellung und Begründung des Forschungsimpacts in der PGF.

Die identifizierten Wirkungen und Wirkungswege von *PartKommPlus* sind auf 3 verschiedenen Ebenen festzustellen: 1) Wirkungen auf individueller und gemeinschaftlicher Ebene, 2) Wirkungen auf Ebene der Praxis und 3) Wirkungen auf Wissenschaftsebene. Wirkungen auf individueller und gemeinschaftlicher Ebene waren bespielhaft in der Form einer wechselseitigen Sensibilisierung gegenüber den Anliegen und Perspektiven der unterschiedlichen Beteiligtengruppen (Wissenschaft, Praxis, Adressat*innen) oder auch als Prozess des Empowerments bei den Beteiligten festzustellen. Wirkungen auf Ebene der Praxis betreffen vor allem einen Wissens- und Kompetenzgewinn hinsichtlich der Umsetzung partizipativer Arbeitsweisen in der kommunalen Gesundheitsförderung. Auf der Wissenschaftsebene konnte *PartKommPlus* Interesse an partizipativer Gesundheitsforschung unter Wissenschaftler*innen wecken bzw. bestärken und Auseinandersetzungen über diesen Forschungsansatz anregen, u. a. im Rahmen gemeinsamer Workshops.

### SMARTACT

Das Ziel von *SMARTACT* (Individuelle und kontextbasierte Interventionen in Echtzeit zur Förderung des normalen Essverhaltens und der körperlichen Aktivität unter Einsatz mobiler Technologie) ist die Entwicklung einer evidenzbasierten Interventionstoolbox zur Förderung des Gesundheitsverhaltens im Bereich der Ernährung und körperlichen Aktivität unter Einsatz mobiler Technologien (Smartphones, Sensoren). Im Sinne des EBPH-Modells legt *SMARTACT* einen Fokus auf die Evidenzgenerierung zu mobilen Anwendungen für eine effektive Primärprävention. Diese werden als vielversprechend eingeschätzt, da sie das Verhalten unmittelbar im Moment des Entstehens erfassen („in the moment“) und personalisierte Interventionen dann darbieten, wenn diese für die Nutzer*innen besonders relevant und hilfreich sind („just in time“; [55, 56]). Da über 80 % der Erwachsenen in Deutschland ein Smartphone besitzen, können mobile, personalisierte Interventionen zur Förderung des Gesundheitsverhaltens einem großen Personenkreis vergleichsweise kostengünstig zur Verfügung gestellt werden. *SMARTACT* entwickelt in 7 Teilprojekten eine modulare Toolbox auf der Grundlage einer gemeinsamen Plattform („Apps in der App“) mit Anwendungen zur Förderung des Ernährungsverhalten und der körperlichen Aktivität sowie für verschiedene Lebenskontexte (Familie, Arbeit).

Die evidenzbasierte Entwicklung und Bewertung digitaler Technologien ist mit mehreren Herausforderungen verbunden. Dazu zählen insbesondere die rasche Entwicklung der Technologie, die Menge und Verarbeitung der Daten inklusive der Datensicherheit sowie die Gestaltung der Personalisierung der Messung und Interventionen [57]. Die Entwicklung der *SMARTACT-*Interventionstools erfolgt auf der Grundlage einer multiphasischen Optimierungsstrategie, die verschiedene Entwicklungsstufen und einen Mixed-Methods-Ansatz umfasst. Für die Entwicklung eines *SMARTACT*-Tools (z. B. *SMARTFOOD*-App [58], *SMARTFAMILY*-App [56]) wurde eine umfassende, partizipative Co-Designstrategie genutzt [59]. Hier wurden im Rahmen von qualitativen und quantitativen Pretests und Pilotstudien mit potenziellen Nutzer*innen die Anwendungen zur Erfassung und Intervention in iterativen Feedbackschlaufen entwickelt und erprobt. In dieser Entwicklungs- und Erprobungsphase wurde durch die Zusammenarbeit von Nutzer*innen und Expert*innen ein erster Prototyp auf der Basis von theoriebasierten und empirischen Forschungsergebnissen erstellt. Grundlage hierfür waren u. a. die Durchführung von systematischen Reviews und Metaanalysen [60] sowie theoretische Konzepte, wie beispielsweise Verhaltensmotive, Hinweisreize, Feedback, auf künstlicher Intelligenz basierende Dialogsysteme und Strategien zur Verhaltensänderung [55, 61]. Die Anwendungen wurden in Bezug auf die Nutzerfreundlichkeit, Funktionalität und Wirksamkeit optimiert, was nur durch eine enge Zusammenarbeit zwischen den potenziellen Anwender*innen und den Expert*innen aus den verschiedenen Teilprojekten und Fachdisziplinen möglich war. Hier konnte mithilfe der *SMARTFOOD*-App, die u. a. eine fotobasierte Erfassung des Essverhaltens umfasst, eine sehr hohe Teilnahme- und Verhaltenserfassungsrate realisiert werden [62].

Mobile Anwendungen ermöglichen auch eine neue Form der evidenzbasierten Partizipation der Nutzer*innen und Forscher*innen. Durch die Entwicklung von Datenvisualisierungen erhielten die Teilnehmer*innen u. a. in Echtzeit Rückmeldungen zu ihrer persönlichen Verhaltenssignatur und die anonymisierten Daten und Ergebnisse konnten einer breiten Öffentlichkeit zur Verfügung gestellt werden (z. B. https://smartexplore.dbvis.de/ [63]). Zusätzlich wurden Immersive Analytics eingesetzt, d. h. Datenvisualisierungen unter Einsatz von Augmented-Reality-Techniken (computergestützte Erweiterung der Realitätswahrnehmung) realisiert, die neue Auswertungsmöglichkeiten der umfassenden Daten der Interventionsstudien ermöglichen [64]. Die Effektivität der neu entwickelten Anwendungen wurde auch im Rahmen von RCTs evaluiert sowie aus einer gesundheitsökonomischen Perspektive analysiert. *SMARTACT* arbeitet regional mit ortsansässigen Kooperationspartner*innen (z. B. Landratsamt, Firmen) zusammen, um die entwickelte *SMARTACT-*Toolbox praxisnah zu erproben, und erreicht ferner über diese Multiplikator*innen einen raschen Ergebnistransfer der Forschungsergebnisse in die Praxis. Die Forschungsarbeiten in *SMARTACT *lassen eine beständige Oszillation zwischen EBPH-Bereichen wie Evidenzgenerierung und kommunikativen Prozessen des Austauschens und Handelns erkennen, die möglicherweise besonders bei neuartigen Maßnahmen, z. B. unter Nutzung digitaler Technologien, sehr intensiv verläuft.

## Möglichkeiten, Grenzen und Perspektiven der Evidenzbasierung – Erkenntnisse der Forschungsverbünde in Primärprävention und Gesundheitsförderung

In Anlehnung an das EBPH-Modell von Gerhardus konnte der umfassende Anspruch an evidenzbasiertes Forschen und Umsetzen ebenso wie die jeweilige Schwerpunktsetzung in den einzelnen Forschungsverbünden dargestellt werden.

### Einbindung von nichtakademischen und zivilgesellschaftlichen Akteur*innen

In der Primärprävention und Gesundheitsförderung gilt es, eine zunehmende Evidenzbasierung zu erreichen und dies mit den besonderen Ansprüchen an Partizipation zu verknüpfen. In den Forschungsverbünden werden daher nichtakademische und zivilgesellschaftliche Akteur*innen (z. B. Fachkräfte aus Prävention und Gesundheitsförderung, Politik, Adressat*innen von Public-Health-Maßnahmen) sowie Nutzer*innen in die Forschungsarbeit mit unterschiedlichem Beteiligungsgrad einbezogen. Einerseits ergibt sich dies aus den oben dargestellten Anforderungen an evidenzbasierte Public-Health-Maßnahmen, andererseits haben die Verbünde gute Erfahrungen mit der Einbindung nichtakademischer, zivilgesellschaftlicher Akteur*innen und Nutzer*innen gemacht, um wirksame Public-Health-Maßnahmen zu entwickeln, zu implementieren und zu evaluieren. Barrieren für die Einbindung nichtakademischer und zivilgesellschaftlicher Akteur*innen und Nutzer*innen stellen fehlende Finanzierungsmöglichkeiten zur Beteiligung an Forschungsanträgen und kurze Förderzeiträume dar [65]. Darüber hinaus konnten nicht alle Verbünde die selbstgesteckten Ziele hinsichtlich der Einbindung vulnerabler Gruppen (Personen mit Migrationshintergrund, Menschen mit niedrigem sozioökonomischen Status) durchgängig erreichen. Damit bleibt auch die Evidenzlage in Bezug auf diese Gruppen z. T. lückenhaft. Eine Ausdehnung der Förderzeiträume würde die jetzigen und zukünftige Forschungsverbünde in die Lage versetzen, nichtakademische, zivilgesellschaftliche Akteur*innen und Nutzer*innen noch besser einzubinden und damit auch hinsichtlich schwierig zu erreichender Gruppen die Evidenzbasierung zu stärken. Dies sollte dann auch verstärkt in Ausschreibungen der Förderer*innen berücksichtigt werden.

### Nachhaltige Sichtbarkeit von Forschungsergebnissen

Mit dem Auslaufen des Förderzeitraums enden meistens auch die Möglichkeiten, die generierten Erkenntnisse für eine Anwendung in der Praxis nachhaltig sichtbar zu halten. Während die Erkenntnisse auf der wissenschaftlichen Ebene langfristig durch wissenschaftliche Publikationen erhalten bleiben, zeigen Ergebnisse aus den Verbünden das Potenzial eines verstärkten Einsatzes transdisziplinärer Ansätze und partizipativer Forschungsmethoden (z. B. kooperative Planung, Co-Design, partizipative Gesundheitsforschung). Verbundübergreifende Strukturen sichern einerseits eine Vernetzung der Verbünde und tragen dazu bei, die Forschungserkenntnisse langfristig für die Praxis und Netzwerke sichtbar zu halten, – so kann neue Evidenz den Weg in den Alltag der Prävention und Gesundheitsförderung finden. Eine gemeinsame Koordination, umgesetzt im Zusammenschluss der 5 Forschungsverbünde im Forschungsnetzwerk Primärprävention und Gesundheitsförderung (FP2G.net), kann mit gemeinsamen Veranstaltungen, Publikationen und Öffentlichkeitsarbeit dazu beitragen, die entwickelte Evidenz in Form von Arbeitsmaterialien und Ergebnisberichten für die Fachöffentlichkeit und Praxis auch längerfristig zur Verfügung zu stellen. Für die Bearbeitung neuer Public-Health-Probleme ist eine gute Zugänglichkeit von Methoden, Daten und Ergebnissen abgeschlossener oder laufender Forschungsaktivitäten ein wichtiger Aspekt, der eine zunehmende Evidenzbasierung in diesem Bereich fördert.

#### Weiterentwicklung der Forschungsmethodik

Die COVID-19-Pandemie verdeutlicht die Abhängigkeit wissenschaftlicher Prozesse von einem stabilen und forschungsförderlichen Umfeld. In der Pandemie war und ist die Forschung zu Prävention und Gesundheitsförderung gefordert, innovative und den (schnell wandelbaren) Rahmenbedingungen angepasste Methoden zu entwickeln und zu evaluieren, um Forschungsfragen bestmöglich bearbeiten zu können. Mehrere Forschungsverbünde waren hierfür in ihren Teilprojekten durch digitale Forschungsansätze recht gut aufgestellt, für andere war die Interaktion von Mensch zu Mensch unumgänglich oder das Forschungssetting verschlossen. Auch die explorative und zeitnahe Entwicklung und Evaluierung von Methoden müssen daher ihren Platz in der EBPH-Forschungspraxis haben, um Evidenzbasierung und die spezifisch notwendige Evidenzentwicklung unter den jeweils realen Bedingungen neu auszutarieren.

## Fazit

Die Forschungsverbünde konnten Public-Health-Evidenz sowohl unter Studienbedingungen als auch unter realen (Alltags‑)Bedingungen und in unterschiedlichen Kontexten erarbeiten. So konnten Grundlagen für neue Public-Health-Maßnahmen geschaffen und die Implementation etablierter Maßnahmen analysiert und verbessert werden. Der intensive Austausch mit der Praxis und innerhalb der Forschungsverbünde erlaubt eine kontinuierliche Reflexion über Wirkung, Nutzen und Potenziale der Forschungsansätze und die sich entwickelnde Evidenzbasis.
